# Consumer Acceptance of Population-Level Intervention Strategies for Healthy Food Choices: The Role of Perceived Effectiveness and Perceived Fairness

**DOI:** 10.3390/nu7095370

**Published:** 2015-09-15

**Authors:** Colin Bos, Ivo Van Der Lans, Frank Van Rijnsoever, Hans Van Trijp

**Affiliations:** 1 Marketing and Consumer Behaviour Group, Wageningen University, Hollandseweg 1, Wageningen 6706 KN, the Netherlands; E-Mails: ivo.vanderlans@wur.nl (I.L.); hans.vantrijp@wur.nl (H.T.); 2 Innovation Studies, Copernicus Institute of Sustainable Development, Utrecht University, Heidelberglaan 2, Utrecht 3584 CS, the Netherlands; E-Mail: f.j.vanrijnsoever@uu.nl

**Keywords:** obesity prevention, intervention strategies, consumer acceptance, effectiveness, fairness, intrusiveness, beverages, snack foods, low-calorie, healthy choices

## Abstract

The present study investigates acceptance of intervention strategies for low-calorie snack choices that vary regarding the effect they have on consumers’ freedom of choice (providing information, guiding choice through (dis)incentives, and restricting choice). We examine the mediating effects of perceived effectiveness and perceived fairness, and the moderating effects of barriers to choose low-calorie snacks and perceived responsibility for food choice. Data was collected through an online survey, involving three waves that were completed over a seven week timespan. Information was collected on barriers and perceived responsibility, and evaluations of a total of 128 intervention strategies with varying levels of intrusiveness that were further systematically varied in terms of source, location, approach/avoidance, type, and severity. A total of 1173 respondents completed all three waves. We found that the effect of intervention intrusiveness on acceptance was mediated by the perceived personal- and societal effectiveness, and the perceived fairness of interventions. For barriers and perceived responsibility, only main effects on intervention-specific beliefs were found. Government interventions were accepted less than interventions by food manufacturers. In conclusion, the present study shows that acceptance of interventions depends on perceptions of personal- and societal effectiveness and fairness, thereby providing novel starting points for increasing acceptance of both existing and new food choice interventions.

## 1. Introduction

Systematic caloric overconsumption is the main driver of increasing overweight and obesity rates in the Western world [1,2]. In the United States caloric intake through meals increased 8% from 1977 to 2006, while intake through snacks more than doubled (130%) [3]. Additionally, in Europe this trend has been detected [4,5]. Snacking, therefore, is believed to be an important contributor to the obesity problem. Since the foods and drinks that are consumed between meals are generally high in calories [6], stimulating people to choose low-calorie over high-calorie snacks can be an effective method to reduce overweight and obesity rates [7].

Policy makers, scientific researchers, and health-care workers have become increasingly interested in intervention strategies that stimulate healthier and more responsible food-choice behavior [8], particularly because consuming high quantities of calories through sugar and fat potentially becomes addictive [9,10]. These intervention strategies, when effective, can reduce the burden of obesity and related non-communicable diseases such as diabetes, cancers, and cardiovascular diseases [11]. Intervention strategies come in many shapes and forms, varying from public health campaigns to a ban of advertising unhealthy foods to children.

The effectiveness of intervention strategies for healthy eating, however, leaves much to be desired. An extensive analysis of the effectiveness of healthy eating policies in Europe shows that the behavioral impact of different types of interventions (e.g., public information campaigns and fiscal measures) is inconclusive, at best [12]. Even healthy eating interventions aimed at specific demographic target groups (e.g., children and the overweight) are known to have only little effect on behavior [13,14,15,16].

To increase effectiveness, scholars stress that intervention strategies need to be accepted by those who are affected by them [17,18]. The level of acceptance affects the degree to which people are prepared to change their behavior. Reactance theory [19] suggests that insufficient acceptance of interventions leads people to be less likely to perform the desired behavior. In contrast, sufficient acceptance elicits rationalization, leading people to be more likely to adopt the intended behavior [20]. In addition, acceptance of interventions is an important condition for its implementation because stakeholders will be reluctant to intervene without public support [21]. Despite its importance, extensive knowledge on the processes underlying acceptance of intervention strategies for food choices is currently lacking [22].

The aim of the present study is to investigate the processes underlying acceptance of intervention strategies for choosing low-calorie over high-calorie snacks. Furthermore, we examine differences in acceptance of interventions that vary regarding the effect they have on consumers’ individual freedom of choice (level of intrusiveness). Hereto, we use Nuffield’s intervention ladder [23], which distinguishes between providing information (low intrusiveness), guiding choice by providing incentives or disincentives (medium intrusiveness), and restricting choice (high intrusiveness). In addition, to explore whether the processes underlying acceptance are robust across snack categories, we distinguish between beverage- and snack food interventions. The following section describes the theoretical background and the hypotheses of the study.

## 2. Theoretical Framework and Hypotheses

Research on processes underlying the acceptance of interventions has shown that two intervention-specific beliefs are particularly important for acceptance: the perceived effectiveness and the perceived fairness [24]. The perceived effectiveness refers to whether people think interventions will be successful in stimulating a particular behavior, thereby differentiating between effectiveness for themselves and for society as a whole. The perceived fairness, on the other hand, refers to the extent to which people perceive interventions to be a fair way of stimulating a particular behavior. This research, however, originates from the transport policy domain and it is, therefore, unclear whether the results generalize to intervention acceptance in other domains.

A qualitative study from Bos *et al.* [25] suggests that also within the food-related domain perceptions of effectiveness and fairness positively influence acceptance of interventions. They found that when people were asked to elaborate on why they accepted some interventions more than others, they often stated reasons that involved the personal- and societal effectiveness, and fairness of interventions. Therefore, we hypothesize that:

**Hypothesis 1a:** There is a positive effect of perceived personal effectiveness on the acceptance of snack choice interventions.

**Hypothesis 1b:** There is a positive effect of perceived societal effectiveness on the acceptance of snack choice interventions.

**Hypothesis 1c:** There is a positive effect of perceived fairness on the acceptance of snack choice interventions.

Literature on acceptance further suggests that the effect on one’s individual freedom of choice is a strong predictor of intervention acceptance. A comprehensive review by Diepeveen *et al.* [18] concludes that acceptance of government interventions to change different health-related behaviors, including food-related behavior, is strongly influenced by their level of intrusiveness. Using Nuffield’s intervention ladder [23], the review demonstrates that less intrusive interventions, of which the main purpose is to provide information, are generally accepted more than intrusive interventions, which mainly aim to restrict choices. This effect of intrusiveness has also been found in choice experiments; Pechey *et al.* [26] conclude that for reducing alcohol intake the least intrusive interventions are most accepted. We therefore hypothesize that:

**Hypothesis 2a:** More intrusive snack choice interventions are less accepted.

Since literature suggests that acceptance is mediated by the perceived effectiveness and perceived fairness [24,25], one would expect an effect of intrusiveness on these intervention-specific beliefs as well. However, in the literature on food-related policies, this effect has received surprisingly limited research attention. To our knowledge, there are only a few studies that relate perceptions of intervention effectiveness and intrusiveness [26,27]. These studies, however, present people with information about the expected effectiveness of interventions and they do not ask people about their perceptions of intervention effectiveness. Therefore, no conclusions can be drawn about the relation between intervention intrusiveness and perceived effectiveness.

In the transport policy context, Eriksson *et al.* [24] found that improving public transport was perceived as more effective than raising taxes on fossil fuels. As raising taxes on fossil fuels obviously is more intrusive than improving public transport, this suggests that there is a relation between intrusiveness and perceptions of effectiveness. To examine whether this relation holds for snack choice interventions, we hypothesize that:

**Hypothesis 2b:** More intrusive snack choice interventions are perceived to be less effective on a personal level.

**Hypothesis 2c:** More intrusive snack choice interventions are perceived to be less effective on a societal level.

In addition, Eriksson *et al.* [24] found that improving public transport was also perceived as fairer than raising taxes on fossil fuels. Within the food-related policy context, Promberger *et al.* [28] examined the perceived fairness of financial interventions *versus* medical interventions. These types of interventions, however, are not easily categorized in terms of level of intrusiveness and therefore the relation between intervention intrusiveness and perceived fairness in a food-related policy context remains unclear. Based on the research in the transport policy domain, we hypothesize that:

**Hypothesis 2d:** More intrusive snack choice interventions are perceived to be less fair.

Connecting the hypotheses about the effect of intrusiveness on perceptions of effectiveness and fairness (2a, 2b, 2c, and 2d) with the hypotheses about the positive effect of the perceived effectiveness and the perceived fairness on acceptance (1a, 1b, and 1c), we come to the overarching hypothesis that:

**Hypothesis 3:** The effect of intrusiveness on acceptance of interventions is mediated by the perceived personal- and societal effectiveness, and the perceived fairness of interventions.

With respect to effectiveness of interventions, Rothschild’s Motivation-Opportunity-Ability (MOA) framework [29] theorizes that it depends on people’s barriers whether an intervention will be effective: people who lack ability need education (equivalent to providing information), people who lack opportunity need marketing (equivalent to guiding choice), and people who lack motivation need law (equivalent to restricting choice). Extrapolating this logic to the present study, we expect that people’s perceived barriers for choosing low-calorie over high-calorie snacks influences the relation between intervention intrusiveness and perceived effectiveness.

Bos *et al.* [30] found three types of barrier profiles for choosing low-calorie snacks over high-calorie snacks: the no-barrier profile, which is characterized by high levels of motivation, opportunity, and ability; the lack-of-opportunity profile, which has a high level of motivation, a low level of opportunity, and a medium level of ability; and the lack-of-motivation profile, which features a low level of motivation and medium levels of opportunity and ability. These profiles applied to both the beverage and snack food categories. Reasoning from the MOA framework, we hypothesize that:

**Hypothesis 4a:** The relation between intervention intrusiveness and the perceived personal effectiveness of interventions is moderated by perceived barriers for choosing low-calorie snacks.

**Hypothesis 4b:** The relation between intervention intrusiveness and the perceived societal effectiveness of interventions is moderated by perceived barriers for choosing low-calorie snacks.

Additionally, we expect that the relation between intervention intrusiveness and perceptions of fairness will be moderated as well. Studies suggest that one’s perception about the responsibility for food choices is related to the perceived fairness of interventions [31,32]. These studies found that the more one believes that responsibility for food choices lie with other stakeholders such as the government and food suppliers, the more they thought interventions to change the food environment were fair. When one felt that the responsibility only lies with people themselves, however, support for more intrusive interventions was low. We therefore hypothesize that:

**Hypothesis 5:** The relation between intervention intrusiveness and the perceived fairness of interventions is moderated by people’s perceptions of personal responsibility for food choices.

**Figure 1:**
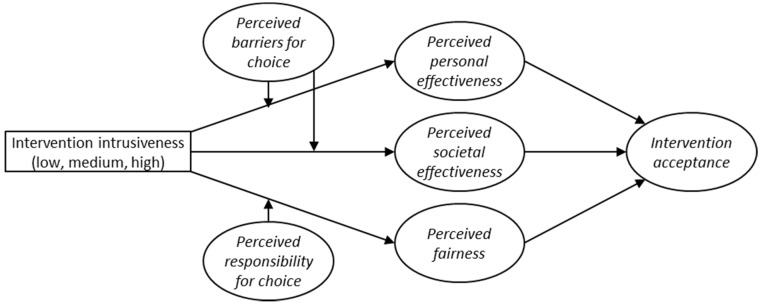
Overview of the present study’s conceptual framework.

By testing the conceptual framework that is depicted in Figure 1, we aim to better understand the processes that underlie acceptance of intervention strategies for low-calorie snack choices as well as the influence of intervention intrusiveness on these processes. With this knowledge policy makers in the food domain can anticipate negative and positive evaluations of new intervention strategies and address these sentiments accordingly.

## 3. Material and Methods

### 3.1. Procedure and Sample

The research protocol for the project “Matching supply and demand side preferences in food innovation” (NWO project number 2100882000), which includes the present article, was submitted to the Social Science Ethics Committee of the Wageningen University and subsequently approved for fulfilling the Wageningen University code of conduct.

An online survey was conducted, comprising of three waves. Wave 1 was divided into two parts, which were presented in a random order: one part focused on individuals’ motivation, opportunity, and ability to choose low-calorie over high-calorie beverages, while the other part focused on the same factors for snack foods. Four weeks later (Wave 2) respondents filled out a Food Frequency Questionnaire (not relevant for the present study) and a questionnaire regarding the perceived responsibility for food choices. Another three weeks later (Wave 3) respondents were asked to rate 16 intervention strategies for stimulating low-calorie snack choices on perceived effectiveness, perceived fairness and acceptance. Respondents were assigned to evaluate either beverages or snack food interventions. Splitting up the data collection in three waves was done to prevent respondent burden and the intermissions were employed to minimize consistency bias.

Data was collected in the Netherlands by GfK (Gesellschaft für Konsumforschung), which is a commercial marketing research agency. A representative sample of the Dutch population based on gender, age, and education level was recruited from the GfK Online Panel. For their participation, respondents received a number of “GfK points” (equivalent to 5 euros), with which they could order coupons. Of the 1573 respondents that completed Wave 1, a total of 1318 completed Wave 2, and 1173 completed all three waves. Response rates were 70%, 84%, and 89%, respectively. Additionally, a new group of 146 respondents completed solely Wave 3, to analyze bias that may have occurred through the completion of Wave 1 and 2. A summary of characteristics of the 1319 respondents can be found in Table 1.

**Table 1:** Respondent characteristics.

	Wave 1, 2, and 3 (*n* = 1173)		Only Wave 3 (*n* = 146)	
	Males (*n* = 559)	Females (*n* = 614)	Males (*n* = 76)	Females (*n* = 70)
Age				
18–34	25.6%	27.4%	27.6%	28.6%
35–49	34.5%	31.2%	34.2%	38.6%
50–65	39.9%	41.4%	38.2%	32.9%
Education ^1^				
Low	26.3%	29.3%	25.0%	27.1%
Middle	41.7%	43.8%	46.1%	40.0%
High	32.0%	26.9%	28.9%	32.9%

^1^ Based on International Standard Classification of Education (ISCED): Low equates to lower-secondary education or less, middle equates to upper-secondary or post-secondary education, and high equates to tertiary education.

### 3.2. Stimuli

The intervention strategies that respondents were confronted with in Wave 3 differed on the level of intrusiveness (providing information *versus* guiding choice *versus* restricting choice). For each level of intrusiveness, intervention strategies were operationalized in multiple ways to check whether our findings with respect to intrusiveness generalize across different implementations. Based on earlier research on acceptance of interventions in the food domain [26,30], operationalizations varied with respect to the following characteristics: 1) the source of the intervention (the government *versus* a group of leading food manufacturers), 2) the location (supermarkets *versus* points-of-purchase on gas- and train stations, and 3) approach-avoidance (encouraging low-calorie choices *versus* discouraging high-calorie choices). Since restricting choice is logically incompatible with encouraging low-calorie-choices, this combination was not offered to respondents.

Intervention strategies were further varied in terms of intervention type and severity level (see Table 2). Providing information was divided into product labeling and information campaigns, guiding choice was divided into financial and assortment (dis)incentives, and restricting choice was divided into restriction of physical choice and restriction of advertising. Similarly, severity was nested with the type of intervention.

**Table 2:** Intervention type and severity level.

Type	Severity
Product labelling	Back-of-pack, Front-of-pack
Information campaigns	On location, On location + media
Financial (dis)incentives	10%, 20%, 30%, 40%
Assortment (dis)incentives	15%, 30%, 45%, 60%
Physical choice restriction ^1^	15%, 30%, 45%, 60%
Advertising restriction ^1^	15%, 30%, 45%, 60%

^1^ Restriction of a % of the most high-calorie snacks

The 128 possible intervention strategies (32 informing, 64 guiding, 32 restricting) were divided into eight blocks with each 16 interventions (four informing, eight guiding, and four restricting). Respondents were randomly assigned to one of the eight blocks. Each block was designed in such a way that within the three levels of intrusiveness, respondents were confronted with every attribute and each level. This way, the blocking factor was un-confounded with the main effects of intrusiveness level and intervention characteristics, as well as with the interaction effects of intrusiveness level and each of the intervention characteristics.

Respondents were presented with a description of the intervention strategy, which was coupled with corresponding icons that illustrated the intervention. The structure of the description was as follows: “Through policy, the government obliges supermarkets to extend shelf space for low-calorie snack foods with 30%.” Above this description, respondents saw icons that illustrated the attributes: a government logo, a supermarket icon, and a representation of shelf space extension with the number 30%.

### 3.3. Measures

#### 3.3.1. Evaluations of Intervention Strategies

Respondents evaluated a total of 16 intervention strategies on four items. These were seven-point Likert-scale items, with labels ranging from “completely disagree” to “completely agree” (coded from 1 to 7 for the analyses), and consisted of the perceived personal- and societal effectiveness (two items: “This strategy would lead me to choose low-calorie over high-calorie beverages/snack foods more often than I currently do” and “This strategy would lead a lot of people to choose low-calorie over high-calorie beverages/snack foods more often than they currently do), the perceived fairness (one item: “I think this strategy is a fair way to encourage people to choose low-calorie over high-calorie beverages/snack foods”), and the acceptance of the interventions (one item: “I support the implementation of this strategy”).

#### 3.3.2. Barriers for Low-Calorie Choices

To map respondents’ barriers we employed Rothschild’s MOA framework [29], which theorizes that both individual-level and environmental determinants drive food-choice behavior. Because the MOA framework itself does not dictate how the Motivation, Opportunity, and Ability constructs should be measured, we used and adapted scales from existing scales from the behavioral theories. In total, 27 items were used for measuring Motivation (12), Opportunity (10), and Ability (five). A detailed description of the construction of the questionnaire can be found in Bos *et al.* [30].

#### 3.3.3. Perceived Personal Responsibility for Food Choice

The measure for perceived personal responsibility for food choice was adapted from Lusk and Ellison [31] and was assessed by one item: “To what extent do you think that the following stakeholders are (partly) responsible for whether people choose low-calorie or high-calorie snacks?” Respondents were asked to distribute 100 points over five stakeholders: the government, food manufacturers, food suppliers other than supermarkets (bakeries, restaurants), supermarkets, and consumers themselves. The final score consisted of the number of points that were allocated to “consumers themselves”.

### 3.4. Data Analysis

To explore whether there is a positive effect of the perceived personal- and societal effectiveness and the perceived fairness on the acceptance of interventions (H1), we regressed acceptance simultaneously on these three constructs by multiple linear regression in SPSS (Version 22) (IBM Corp., Armonk, NY, USA). For all the variables in this analysis we used respondents’ mean-centered scores to cancel out individual differences in mean acceptance and to make it reasonable that the errors will be uncorrelated [33].

To investigate the effect sizes of intrusiveness on perceptions of personal- and societal effectiveness, fairness, and acceptance (H2), and the moderating effects of barriers for low-calorie choices and the perceived personal responsibility for food choice (H4 and H5), we performed an analysis of (co)variance in SPSS. For this analysis we used respondents’ mean-centered scores. We restricted our interpretation of results to only those (large enough) effects with a partial *η*^2^ > 0.015.

To provide information on respondents’ absolute evaluations of interventions, we report the means of the perceived personal- and societal effectiveness, the perceived fairness, and acceptance in terms of the original scale (not mean-centered) to interpret the direction of the effects. Within each analysis of variance, we ran *post hoc* comparisons using the Games-Howell procedure to control the family-wise error rate.

To explore whether the effect of intrusiveness on acceptance is mediated by the perceived personal- and societal effectiveness, and the perceived fairness (H3), we added these intervention-specific beliefs into the analysis of (co)variance. To analyze mediation effects, we followed the steps for testing mediational hypotheses, as presented by Baron and Kenny [34].

To explore direct (between-subjects) effects of barriers on absolute perceptions of intervention effectiveness, we used original (not mean-centered) scores to identify differences between the three dominant barrier profiles regarding perceptions of effectiveness. Profiles of barriers were identified by means of cluster analysis. An extensive description of the data analysis and the barrier profiles that were identified can be found in Bos *et al.* [30].

Additionally, we ran mixed model variants of the regression analysis and the analyses of (co)variance on the original (not mean-centered) data (using a random intercept). These mixed models are another way of controlling for possibly-correlated error terms due to multiple observations from each respondent. Within the mixed models, we used questionnaire version as a blocking factor, to rule out that this influenced the results. We found that the *p*-values for the mixed model analyses were very similar to the *p*-values of the mean-centered data analyses, and therefore did not lead to different conclusions. Questionnaire version did not have a significant effect in any of the mixed model analyses. For clarity purposes, we only report the results of the analyses with the mean-centered data.

## 4. Results

Prior to testing Hypothesis 1a, 1b, and 1c, we closely inspected correlations between the perceived personal- and societal effectiveness, the perceived fairness, and acceptance of beverage- and snack food interventions (Table 3). There were positive correlations between perceived personal- and societal effectiveness and acceptance (*r* = 0.57–0.63) and a strong positive correlation between perceived fairness and acceptance (*r* = 0.82). Correlations between perceived personal- and societal effectiveness and perceived fairness were also positive (*r* = 0.54–0.69). For the remainder of the results section we will use the overarching term “snack choice interventions” if results are similar for beverage- and snack food interventions.

**Table 3:** Correlations between intervention-specific beliefs and acceptance of beverage interventions (upper part) and snack food interventions (lower part).

Intervention Evaluations	Personal Effectiveness	Societal Effectiveness	Fairness	Acceptance
Personal effectiveness	1.00	0.69 ***	0.54 ***	0.57 ***
Societal effectiveness	0.67 ***	1.00	0.57 ***	0.59 ***
Fairness	0.55 ***	0.58 ***	1.00	0.82 ***
Acceptance	0.59 ***	0.63 ***	0.82 ***	1.00

*** *P* < 0.001

Subsequently, a multiple linear regression analysis of acceptance on the perceived personal- and societal effectiveness, and the perceived fairness of snack choice interventions was performed. Variance Inflation Factor (VIF) values were <3, indicating that multi-collinearity did not pose a problem. The intervention-specific beliefs accounted for 71% of the variance in acceptance of snack choice interventions. Perceptions of personal- (β = 0.115) and societal effectiveness (β = 0.144), and fairness (β = 0.679) had a significant positive effect on acceptance of interventions. This indicates that there was a substantial individual effect of each intervention-specific belief on acceptance, even though they were positively correlated. We, therefore, accept Hypothesis 1a, 1b, and 1c and conclude that there is a positive effect of the perceived personal- and societal effectiveness, and the perceived fairness on the acceptance of interventions that stimulate low-calorie snack choices.

Table 4 shows the results from analyses of variance of intrusiveness and intervention attributes on the perceived personal- and societal effectiveness, the perceived fairness, and acceptance of snack choice interventions. To check whether completing Waves 1 and 2 influenced Wave 3 evaluations, we compared the effects of intrusiveness on intervention-specific beliefs and acceptance with the same effects in the new group of respondents (*n* = 146). Only for perceived fairness a significant difference (*p* = 0.025) was found. The effect size, however, was negligibly low (partial *η*^2^ = 0.001). The results indicate that the level of intrusiveness influenced both the perceived fairness and acceptance (partial *η*^2^ = 0.016–0.030). In contrast, the level of intrusiveness did not influence perceptions of personal- and societal effectiveness (partial *η*^2^ = 0.000–0.002); therefore we reject Hypothesis 2b and 2c.

The directions of the main effects of intrusiveness on the perceived fairness and acceptance can be derived from Table 5 (beverage interventions) and Table 6 (snack food interventions). Hypothesis 2a is confirmed, because more intrusive snack choice interventions are indeed less accepted. Similarly, more intrusive interventions are perceived as less fair, thereby confirming Hypothesis 2d.

With respect to intervention attributes, we see in Table 4 that location, type, and severity either have no or a negligibly small effect on the evaluations of interventions (partial *η*^2^ = 0.000–0.013). For source (partial *η*^2^ = 0.007–0.031) and approach/avoidance (partial *η*^2^ = 0.022–0.054), however, evaluations of their respective levels were different. The directions of the main effects of source, approach/avoidance, and the interactions of intrusiveness with source and approach/avoidance can be found in Table 5 and Table 6.

We did not develop specific hypotheses about different effects of attributes on evaluations of interventions. Still, we found that interventions that encourage low-calorie choices were perceived as more effective, fairer, and more acceptable than those that discourage high-calorie choices. This only applied to interventions that guide choice through (dis)incentives; for interventions that provide information, encouragement, and discouragement were evaluated similarly. As restricting choice only applies to discouraging high-calorie choices, no effects can be reported.

A similar pattern emerged for source: interventions implemented by the government were perceived as less fair and were also less accepted than interventions implemented by a group of leading food manufacturers. This was true for interventions that guide choice and interventions that restrict choice. Again, for providing information evaluations were similar for both sources.

Table 7 shows the results from the analysis of variance with and without adding the intervention-specific beliefs to the model, to explore whether they mediate the effects of intrusiveness and intervention attributes on acceptance of snack choice interventions. Compared to the effect sizes without adding intervention-specific beliefs, the effects of intrusiveness and intervention attributes on acceptance almost disappeared. Subsequently, we performed Sobel tests [35] to test the mediating effect of each intervention-specific belief on the relation between each level of intrusiveness and acceptance. All Sobel tests yielded significant mediation effects (*p* < 0.001). In support of Hypothesis 3, we conclude that the effect of intrusiveness on acceptance of interventions is mediated by the perceived personal- and societal effectiveness, and the perceived fairness of interventions.

**Table 4:** Effect sizes (partial *η*^2^) of intrusiveness, intervention attributes, barrier profile, and responsibility on evaluations of interventions.

Main- and Interaction Effects	Beverage Interventions ^a^				Snack Food Interventions ^a^			
	Personal Effectiveness	Societal Effectiveness	Fairness	Acceptance	Personal Effectiveness	Societal Effectiveness	Fairness	Acceptance
Intrusiveness (H2)	0.002 **	0.000	**0.030** ***	**0.016** ***	0.000	0.000	**0.028** ***	**0.016** ***
*Intervention attributes*								
Source	0.007 ***	0.009 ***	**0.025** ***	**0.024** ***	0.007 ***	0.007 ***	**0.031** ***	**0.030** ***
Location	0.002 ***	0.002 ***	0.000	0.000	0.003 ***	0.001*	0.000	0.000
Approach/avoidance	**0.022** ***	**0.023** ***	**0.043** ***	**0.047** ***	**0.029** ***	**0.028** ***	**0.051** ***	**0.054** ***
Type within								
Low intrusiveness	0.000	0.000	0.000	0.001 **	0.000	0.001 **	0.000	0.000
Medium intrusiveness	0.012 ***	0.009 ***	0.001 *	0.000	0.003	0.002 ***	0.003 ***	0.001 *
High intrusiveness	0.001 **	0.002 ***	0.004 ***	0.003 ***	0.000	0.000	0.013 ***	0.013 ***
Severity	0.005 ***	0.008 ***	0.000	0.001*	0.003 ***	0.008 ***	0.000	0.001
Intrusiveness × attributes ^1^	0.010 ***	0.013 ***	0.026 ***	0.025 ***	0.011 ***	0.013 ***	0.033 ***	0.031 ***
*Moderating effect of barrier profile (H4)*	0.000	0.001	-	0.002 **	0.002 ***	0.002 ***	-	0.001
Barrier profile × intrusiveness	0.000	0.001	-	0.002 **	0.002 ***	0.002 ***	-	0.001
Barrier profile × attributes ^2^	0.002	0.000	-	0.003	0.002	0.003	-	0.002
Barrier profile × intrusiveness × attributes ^3^	0.003	0.004	-	0.005	0.003	0.002	-	0.001
*Moderating effect of perceived responsibility (H5)*								
Responsibility × intrusiveness	-	-	0.002 ***	0.001 *	-	-	0.004 ***	0.003 ***
Responsibility × attributes ^2^	-	-	0.006	0.006	-	-	0.001	0.002
Responsibility × intrusiveness × attributes ^3^	-	-	0.000	0.000	-	-	0.003	0.002
Total *R*^2^ (adjusted *R*^2^)	0.073 (0.067)	0.070 (0.064)	0.147 (0.143)	0.133 (0.126)	0.069 (0.063)	0.069 (0.062)	0.164 (0.160)	0.155 (0.148)

* *p* < 0.05, ** *p* < 0.01, *** *p* < 0.001. **Bold numbers** indicate an effect size > 0.015. ^1^ The sum of interactions of intrusiveness with source, location, approach/avoidance, and severity. ^2^ The sum of interactions of barrier profile/responsibility with each of the five attributes; ^3^ The sum of interactions of barrier profile/responsibility with each of the three levels of intrusiveness and each of the five attributes; ^a^ If Levene’s test was significant, we performed bootstrapping. Bootstrap-based standard errors for the estimated marginal means were similar to those from the original analyses.

**Table 5:** Means of the effect sizes (partial *η*^2^ > 0.015) for beverage interventions.

Main- and Interaction Effects	Personal Effectiveness		Societal Effectiveness		Fairness		Acceptance	
	Mean	SD	Mean	SD	Mean	SD	Mean	SD
**Intrusiveness and Intrusiveness × Type**								
Inform choice (low)	4.12 ^y^	1.65	4.57 ^y^	1.45	4.94 ^z^	1.45	4.86 ^z^	1.54
Product labelling	4.17 ^c,d^	1.66	4.60 ^c,d^	1.48	4.95 ^c^	1.46	4.93 ^c^	1.55
Information campaigns	4.08 ^b,c^	1.63	4.56 ^b,c^	1.43	4.93 ^c^	1.45	4.78 ^c^	1.53
Guide choice (medium)	4.18 ^y^	1.70	4.58 ^y^	1.51	4.37 ^y^	1.65	4.47 ^y^	1.71
Financial (dis)incentives	4.34	1.72	4.74 ^d^	1.53	4.34 ^b^	1.73	4.47 ^b^	1.78
Assortment (dis)incentives	4.02 ^a,b^	1.64	4.45 ^a,b^	1.47	4.42 ^b^	1.57	4.47 ^b^	1.62
Restrict choice (high)	3.90 ^x^	1.68	4.42 ^x^	1.54	4.19 ^x^	1.70	4.29 ^x^	1.76
Physical choice restriction	3.96 ^a^	1.70	4.51 ^a,b,c^	1.56	4.06 ^a^	1.74	4.18 ^a^	1.79
Advertising restriction	3.85 ^a^	1.64	4.34 ^a^	1.52	4.34 ^b^	1.63	4.42 ^b^	1.69
**Source and Source × Intrusiveness**								
Government	4.02 ^x^	1.70	4.45 ^x^	1.55	4.29 ^x^	1.70	4.34 ^x^	1.76
Inform choice	4.10 ^b^	1.64	4.57 ^b,c^	1.47	4.89 ^d^	1.47	4.81 ^d,e^	1.56
Guide choice	4.11 ^b^	1.72	4.50 ^b^	1.56	4.19 ^b^	1.70	4.28 ^b^	1.77
Restrict choice	3.75 ^a^	1.69	4.25 ^a^	1.59	3.88 ^a^	1.74	3.98 ^a^	1.82
Food manufacturers	4.18 ^y^	1.66	4.64 ^y^	1.45	4.66 ^y^	1.56	4.71 ^y^	1.60
Inform choice	4.15 ^a^	1.65	4.59 ^b,c^	1.44	4.98 ^d^	1.44	4.89 ^e^	1.53
Guide choice	4.25 ^c^	1.66	4.69 ^c^	1.45	4.57 ^c^	1.58	4.65 ^c,d^	1.62
Restrict choice	4.06 ^b^	1.64	4.59 ^b,c^	1.47	4.52 ^c^	1.58	4.62 ^c^	1.60
**Approach/avoidance and Appr./avoid. × Intrusiveness**								
Encourage low-calorie choices	4.33 ^y^	1.67	4.77 ^y^	1.44	4.85 ^y^	1.49	4.90 ^y^	1.55
Inform choice	4.18 ^c,d^	1.64	4.61 ^c^	1.46	4.97 ^d^	1.43	4.91 ^c^	1.53
Guide choice	4.41 ^d^	1.68	4.85 ^d^	1.42	4.79 ^c^	1.52	4.89 ^c^	1.55
Restrict choice	-	-	-	-	-	-	-	-
Discourage high-calorie choices	3.95 ^x^	1.67	4.41 ^x^	1.53	4.25 ^x^	1.68	4.30 ^x^	1.73
Inform choice	4.07 ^b,c^	1.66	4.54 ^b,c^	1.46	4.25 ^x^	1.48	4.79 ^c^	1.55
Guide choice	3.95 ^a,b^	1.67	4.34 ^a^	1.55	3.97	1.68	4.05 ^a^	1.75
Restrict choice	3.90 ^a^	1.67	4.42 ^a,b^	1.54	4.20 ^b^	1.69	4.30 ^b^	1.74

^x,y,z^ Superscripts for post-hoc comparisons for main effects. ^a,b,c,d,e^ Superscripts for post-hoc comparisons for interaction effects. Dissimilar superscript letters within rows indicate significant differences, based on Games-Howell post hoc test (*p* < 0.05). Superscripts apply to a single column and single attribute part.

**Table 6:** Means of the effect sizes (partial *η*^2^ > 0.015) for snack food interventions.

Main- and Interaction Effects	Personal Effectiveness		Societal Effectiveness		Fairness		Acceptance	
	Mean	SD	Mean	SD	Mean	SD	Mean	SD
**Intrusiveness and Intrusiveness × Type**								
Inform choice (Low)	4.13 ^y^	1.66	4.44 ^x^	1.49	4.98 ^z^	1.51	4.93 ^z^	1.57
Product labelling	4.16 ^b^	1.72	4.38 ^a,b^	1.55	4.99 ^d^	1.55	4.95 ^c^	1.62
Information campaigns	4.10 ^b^	1.61	4.50 ^b^	1.43	4.96 ^d^	1.49	4.91 ^c^	1.52
Guide choice (medium)	4.19 ^y^	1.71	4.55 ^y^	1.50	4.42 ^y^	1.72	4.51 ^y^	1.75
Financial (dis)incentives	4.27 ^c^	1.75	4.61 ^b^	1.54	4.34 ^b^	1.80	4.47 ^b^	1.83
Assortment (dis)incentives	4.11 ^b^	1.66	4.49 ^b^	1.46	4.51 ^c^	1.64	4.54 ^b^	1.66
Restrict choice (high)	3.96 ^x^	1.69	4.35 ^x^	1.56	4.27 ^x^	1.72	4.33 ^x^	1.78
Physical choice restriction	4.00 ^a,b^	1.72	4.38 ^a,b^	1.61	4.00 ^a^	1.78	4.07 ^a^	1.84
Advertising restriction	3.81 ^a^	1.66	4.22 ^a^	1.50	4.53 ^c^	1.64	4.59 ^b^	1.67
**Source and Source × Intrusiveness**								
Government	4.03 ^x^	1.72	4.38 ^x^	1.54	4.31 ^x^	1.76	4.35 ^x^	1.79
Inform choice	4.11 ^b^	1.68	4.44 ^b^	1.49	4.94 ^d^	1.53	4.89 ^d^	1.61
Guide choice	4.09 ^b^	1.73	4.45 ^b^	1.54	4.20 ^b^	1.78	4.28 ^b^	1.81
Restrict choice	3.81 ^a^	1.72	4.18 ^a^	1.61	3.89 ^a^	1.75	3.97 ^a^	1.82
Food manufacturers	4.21 ^y^	1.67	4.56 ^y^	1.48	4.73 ^y^	1.60	4.78 ^y^	1.63
Inform choice	4.15 ^b,c^	1.65	4.43 ^b^	1.49	5.01 ^d^	1.49	4.97 ^d^	1.53
Guide choice	4.29 ^c^	1.68	4.65 ^c^	1.46	4.64 ^c^	1.63	4.73 ^c,d^	1.53
Restrict choice	4.10 ^b^	1.66	4.52 ^b,c^	1.49	4.64 ^c^	1.62	4.69 ^c^	1.66
**Approach/avoidance and Appr./avoid. × Intrusiveness**								
Encourage low-calorie choices	4.37 ^y^	1.67	4.72 ^y^	1.43	4.92 ^y^	1.54	4.98 ^y^	1.55
Inform choice	4.18 ^c^	1.64	4.49 ^b^	1.46	5.01 ^c^	1.49	4.98 ^c^	1.55
Guide choice	4.47 ^d^	1.68	4.83 ^c^	1.40	4.88 ^c^	1.56	4.98 ^c^	1.56
Restrict choice	-	-	-	-	-	-	-	-
Discourage high-calorie choices	3.96 ^x^	1.69	4.32 ^x^	1.54	4.28 ^x^	1.73	4.32 ^x^	1.78
Inform choice	4.08 ^b,c^	1.69	4.38 ^a,b^	1.52	4.95 ^c^	1.53	4.88 ^c^	1.59
Guide choice	3.91 ^a^	1.69	4.27 ^a^	1.54	3.96 ^a^	1.74	4.03 ^a^	1.80
Restrict choice	3.96 ^a,b^	1.70	4.35 ^a^	1.56	4.27 ^b^	1.72	4.33 ^b^	1.78

^x,y,z^ Superscripts for post-hoc comparisons for main effects. ^a,b,c,d^ Superscripts for *post hoc* comparisons for interaction effects. Dissimilar superscript letters within rows indicate significant differences, based on Games-Howell post hoc test (*p* < 0.05). Superscripts apply to a single column and single attribute part.

**Table 7:** Effect sizes (partial *η*^2^) of intrusiveness and intervention attributes on acceptance of interventions, with (left column) and without (right column) adding intervention-specific beliefs to the model (snack foods and beverages combined).

Main- and Interaction Effects	Partial *η*^2^	Partial *η*^2^
*Intervention-specific beliefs*		
Personal effectiveness	0.023 ***	
Societal effectiveness	0.035 ***	
Fairness	0.447 ***	
Intrusiveness	0.001 ***	0.016 ***
Source	0.003 ***	0.027 ***
Location	0.000	0.000
Approach/avoidance	0.005 ***	0.050 ***
Type	0.001 **	0.001 ***
Severity	0.000	0.000
Intrusiveness × attributes ^1^	0.007	0.032***
Total *R*^2^	0.717 (0.716)	0.127 (0.126)

* *p* < 0.05, ** *p* < 0.01, *** *p* < 0.001. ^1^ The sum of interactions of intrusiveness with source, location, approach/avoidance, and severity.

Hypothesis 4a and 4b posit that the relation between intervention intrusiveness and the perceived personal- and societal effectiveness of interventions is moderated by perceived barriers for choosing low-calorie snacks. Table 4 shows that for both perceptions of effectiveness there was either no or a negligible interaction effect of barrier profile and intrusiveness. We, therefore, reject Hypothesis 4a and 4b.

As literature that relates barriers to effectiveness of interventions strongly suggests that there is a relation [29,30], we also explored direct effects of barrier profile on perceptions of intervention effectiveness. Using original (not mean-centered) perceived effectiveness scores, we did find a direct effect of barrier profile on perceptions of personal- and societal effectiveness (*F*(2, 1168) = 30.75, *p* < 0.001, and *F*(2, 1168) = 17.12, *p* < 0.001). The lack-of-motivation profile consistently scored lowest on the perceived personal- and societal effectiveness for each level of intrusiveness. The lack-of-opportunity profile perceived all levels of intrusiveness to be more personally effective than the no-barrier profile; with respect to perceptions of societal effectiveness these profiles did not differ. The same patterns emerged for perceived fairness (*F*(2, 1168) = 28.99, *p* < 0.001) and acceptance (*F*(2, 1168) = 34.20, *p* < 0.001).

Lastly, we hypothesized that perceptions of personal responsibility for food choice moderate the relation between intervention intrusiveness and the perceived fairness of interventions. Again, we found only a small interaction effect of responsibility and intrusiveness on perceptions of fairness (partial *η*^2^ = 0.002–0.004), and thus we reject Hypothesis 5. We also explored the direct effect of perceptions of personal responsibility on the perceived fairness of interventions using original (not mean-centered) scores. Again, there was a significant but only small effect (*p* < 0.001; partial *η*^2^ = 0.008).

## 5. Discussion

The present study investigated the processes underlying acceptance of intervention strategies for low-calorie snack choices and examined differences in acceptance of interventions with varying levels of intrusiveness. A total of 128 systematically varied intervention strategies were evaluated with respect to three intervention-specific beliefs (perceived personal- and societal effectiveness and the perceived fairness) and overall acceptability.

We found that perceptions of personal- and societal effectiveness, and fairness positively influence acceptance of interventions for low-calorie snack choices. This is in line with the findings from the transport policy domain [24] and also confirms the findings from a qualitative study on acceptance of interventions in the food-choice domain [25]. Our results are robust across the application areas of beverages and snack foods, thereby implying that the relation between intervention-specific beliefs and acceptance is generalizable across food-related product categories.

Perceived fairness emerged as the strongest predictor of intervention acceptance. While being related to perceived fairness, the perceived personal- and societal effectiveness both had unique effects on acceptance as well. This indicates that despite their considerable overlap, both types of intervention-specific beliefs should be considered when maximizing the acceptance of food choice interventions.

In line with literature on changing health-related behaviors [26], we show a greater acceptance of less intrusive interventions (e.g., product labelling). However, the present study extends this literature by showing that the effect of intrusiveness on acceptance is mediated by perceived personal- and societal effectiveness, and perceived fairness of interventions. Herewith, we provide insight into processes underlying acceptance and furthermore show that low levels of perceived effectiveness and perceived fairness form barriers for acceptance. Particularly for intrusive interventions, this finding provides novel starting points for increasing acceptance.

There seems to be a discrepancy between perceptions of effectiveness and actual effectiveness of interventions. While research shows that more intrusive interventions are more effective [16,36], we found that perceptions of personal- and societal effectiveness of providing information (low intrusiveness) and guiding choice (medium intrusiveness) were higher than for restricting choice (high intrusiveness). This could be due to reactance, which occurs when people are feeling restricted in their freedom of choice [19].

The variations in operationalization at the different levels of intrusiveness mostly had very limited, if any, effect on the evaluations of interventions. It was seen as irrelevant on which of the two locations the intervention was implemented (supermarkets *versus* points-of-purchase on gas- and train stations), with which of the two intervention types within the level of intrusiveness this was executed (product labeling *versus* information campaigns; financial (dis)incentives *versus* assortment (dis)incentives; physical choice restriction *versus* advertising restriction), and how strong it attempted to do so. We did find that people perceived interventions that discourage high-calorie choices to be less effective than strategies that encourage low-calorie choices. Literature, however, suggests that interventions that discourage bad choices are, in fact, more effective [37,38]. Some scholars therefore propose that knowledge about intervention effectiveness should be disseminated clearly to the public in order to increase acceptance [26,27].

Interestingly, our study also revealed that perceptions of fairness and acceptance were lower for interventions implemented by the government, as compared to food manufacturers. This could be explained by people perceiving intrusive government interventions to be paternalistic [25], while similar interventions by food manufacturers are probably seen as a natural mechanism of the free market. Low acceptance of government interventions could pose problems for implementation, which would be a delicate matter since experts in the area of food policies stress that governments have a significant role to play in stimulating healthy eating behaviors [39,40]. More research on ways to increase acceptance of intrusive government interventions thus is required.

Contrary to the expectations, we did not find the hypothesized moderating effect of the barrier profile of the “receiver of the intervention” on the relation between intrusiveness and perceived effectiveness. We did find, however, that people with the no-barrier and lack-of-opportunity profiles perceived interventions of all three intrusiveness levels to be more effective, more fair and, therewith, more acceptable than those with the lack-of-motivation profile. Since both the no-barrier and the lack-of-opportunity profile are characterized by high levels of motivation to choose low-calorie snacks, this suggest that the strength of the motivation to behave has an effect on perceptions of effectiveness and fairness. This is in line with what has been found in an earlier study by Eriksson *et al.* [24].

Where food policy research suggests that the perceived personal responsibility moderates the effect of intrusiveness on perceived fairness and acceptance of interventions [31,32], the present study only found a negligibly small interaction effect. The results did show a larger, but still small direct effect, which indicates that people who attribute responsibility for food choice to factors outside personal control perceive interventions that guide and restrict choice to be more fair and acceptable, as compared to people who see food choice mainly as a personal responsibility. This direct effect of perceived responsibility on acceptance of more intrusive interventions has been implied in literature as well [41,42].

The present study comes with a few limitations. As the type of data does not allow one to establish causality, it is possible that causality between intervention acceptance and perceptions of effectiveness and fairness runs in the opposite direction of what is suggested by our model in Figure 1, or even be bi-directional. It could for instance be that when people initially have a positive feeling towards an intervention, they are more likely to perceive this intervention to be more effective as well. While the proposed model is grounded in earlier research on acceptance of interventions, experimental studies are needed to rule out different relations between the intervention evaluations.

Even though we used the Nuffield intervention ladder [23], which specifically ranks interventions according to their intrusiveness, it is possible that respondents’ perceptions of intrusiveness were different. In particular ‘restricting advertising’ was evaluated as a relatively acceptable intervention, which could indicate that people perceive this as a less intrusive intervention that guides rather than restricts choice. In general, however, our results show that interventions that are classified as more intrusive in the Nuffield ladder were accepted less. As this is in line with earlier research that shows when people perceive interventions to be intrusive, they are less likely to accept them [20], this suggests that the objective and subjective intrusiveness likely are quite similar. Since, to our knowledge, no research has been done on perceptions of intervention intrusiveness, future research should encompass the assessment of perceptions of intervention intrusiveness as well.

As the present study was primarily concerned with examining if and how perceptions of effectiveness and fairness predicted acceptance across a wide range of intervention strategies, we assessed these constructs with single items. Although it is common to measure intervention-specific beliefs this way [24,28], it is possible that people differ in how they construct perceptions of effectiveness and fairness of interventions [25]. Future research should therefore look into different connotations of intervention-specific beliefs and examine their unique effects on acceptance.

The present study elicited evaluations of hypothetical strategies to stimulate low-calorie snack choices. Research suggest that the stage of implementation of an intervention (*i.e.*, the certainty that an intervention will be implemented) influences evaluations drastically [18,20]. Therefore it would be valuable to see if and how snack choice interventions in various stages of implementation (e.g., implementation 20% certain *versus* 80% certain) are evaluated differently than the hypothetical strategies in the present study.

## 6. Conclusions

Perceptions of personal- and societal effectiveness, and fairness, influence acceptance of interventions that stimulate healthy food choices. Particularly when interventions are intrusive, it is important for policy makers to address these intervention-specific beliefs to maximize intervention acceptance. We encourage future research on food choice interventions to build upon the present study’s results, thereby further unravelling the processes underlying intervention acceptance.

## References

[1] Shelley J.J. (2012). Addressing the policy cacophony does not require more evidence: An argument for reframing obesity as caloric overconsumption. BMC Public Health.

[2] Swinburn B.A., Sacks G., Hall K.D., McPherson K., Finegood D.T., Moodie M.L., Gortmaker S.L. (2011). Obesity 1 the global obesity pandemic: Shaped by global drivers and local environments. Lancet.

[3] Popkin B.M., Duffey K.J. (2010). Does hunger and satiety drive eating anymore? Increasing eating occasions and decreasing time between eating occasions in the United States. Am. J. Clin. Nutr..

[4] Gracia A., Albisu L.M. (2001). Food consumption in the European Union: Main determinants and country differences. Agribusiness.

[5] Ovaskainen M.L., Reinivuo H., Tapanainen H., Hannila M.L., Korhonen T., Pakkala H. (2005). Snacks as an element of energy intake and food consumption. Euro. J. Clin. Nutr..

[6] Zizza C., Siega-Riz A.M., Popkin B.M. (2001). Significant increase in young adults’ snacking between 1977–1978 and 1994–1996 represents a cause for concern!. Prev. Med..

[7] Cohen D.A., Sturm R., Scott M., Farley T.A., Bluthenthal R. (2010). Not enough fruit and vegetables or too many cookies, candies, salty snacks, and soft drinks?. Pub. Health Rep..

[8] Gortmaker S.L., Swinburn B.A., Levy D., Carter R., Mabry P.L., Finegood D.T., Huang T., Marsh T., Moodie M.L. (2011). Changing the future of obesity: Science, policy, and action. Lancet.

[9] Meule A., Gearhardt A. (2014). Food addiction in the light of DSM-5. Nutrients.

[10] Avena N.M., Rada P., Hoebel B.G. (2008). Evidence for sugar addiction: Behavioral and neurochemical effects of intermittent, excessive sugar intake. Neurosci. Biobehav. Rev..

[11] Traill W.B., Shankar B., Brambila-Macias J., Bech-Larsen T., Aschemann-Witzel J., Strand M., Mazzocchi M., Capacci S., Verbeke W., Perez-Cueto F.J.A. (2010). Interventions to promote healthy eating habits: Evaluation and recommendations. Obes. Rev..

[12] Capacci S., Mazzocchi M., Shankar B., Macias J.B., Verbeke W., Perez-Cueto F.J.A., Koziol-Kozakowska A., Piorecka B., Niedzwiedzka B., D’Addesa D. (2012). Policies to promote healthy eating in Europe: A structured review of policies and their effectiveness. Nutr. Rev..

[13] Kesten J.M., Griffiths P.L., Cameron N. (2011). A systematic review to determine the effectiveness of interventions designed to prevent overweight and obesity in pre-adolescent girls. Obes. Rev..

[14] Maes L., van Cauwenberghe E., van Lippevelde W., Spittaels H., de Pauw E., Oppert J.-M., van Lenthe F.J., Brug J., de Bourdeaudhuij I. (2012). Effectiveness of workplace interventions in Europe promoting healthy eating: A systematic review. Eur. J. Public Health.

[15] Paul-Ebhohimhen V., Avenell A. (2008). Systematic review of the use of financial incentives in treatments for obesity and overweight. Obes. Rev..

[16] Brambila-Macias J., Shankar B., Capacci S., Mazzocchi M., Perez-Cueto F.J.A., Verbeke W., Trail W.B. (2011). Policy interventions to promote healthy eating: A review of what works, what does not, and what is promising. Food Nutr. Bull..

[17] Kelly B., Hughes C., Chapman K., Louie J.C.Y., Dixon H., Crawford J., King L., Daube M., Slevin T. (2009). Consumer testing of the acceptability and effectiveness of front-of-pack food labelling systems for the Australian grocery market. Health Promot. Int..

[18] Diepeveen S., Ling T., Suhrcke M., Roland M., Marteau T.M. (2013). Public acceptability of government intervention to change health-related behaviours: A systematic review and narrative synthesis. BMC Pub. Health.

[19] Brehm J.W. (1966). A Theory of Psychological Reactance.

[20] Laurin K., Kay A.C., Fitzsimons G.J. (2012). Reactance *versus* rationalization: Divergent responses to policies that constrain freedom. Psychol. Sci..

[21] Schuitema G., Steg L., van Kruining M. (2011). When are transport pricing policies fair and acceptable?. Soc. Justice Res..

[22] Mazzocchi M., Cagnone S., Bech-Larsen T., Niedźwiedzka B., Saba A., Shankar B., Verbeke W., Traill W.B. (2014). What is the public appetite for healthy eating policies? Evidence from a cross-European survey. Health Econom Policy Law.

[23] Nuffield Council on Bioethics (2007). Public health: Ethical issues.

[24] Eriksson L., Garvill J., Nordlund A.M. (2008). Acceptability of single and combined transport policy measures: The importance of environmental and policy specific beliefs. Transp. Res. Part a-Policy Pract..

[25] Bos C., van der Lans I.A., van Rijnsoever F.J., van Trijp H.C. (2013). Understanding consumer acceptance of intervention strategies for healthy food choices: A qualitative study. BMC Pub. Health.

[26] Pechey R., Burge P., Mentzakis E., Suhrcke M., Marteau T.M. (2014). Public acceptability of population-level interventions to reduce alcohol consumption: A discrete choice experiment. Soc. Sci. Med..

[27] Promberger M., Dolan P., Marteau T.M. (2012). “Pay them if it works”: Discrete choice experiments on the acceptability of financial incentives to change health related behaviour. Soc. Sci. Med..

[28] Promberger M., Brown R.C.H., Ashcroft R.E., Marteau T.M. (2011). Acceptability of financial incentives to improve health outcomes in UK and US samples. J. Med. Ethics.

[29] Rothschild M.L. (1999). Carrots, sticks, and promises: A conceptual framework for the management of public health and social issue behaviors. J. Mark..

[30] Bos C., van der Lans I., van Rijnsoever F., van Trijp H. (2015). Heterogeneity in barriers regarding the motivation, the opportunity, and the ability to choose low-calorie snack foods and beverages: Associations with real-life choices. Pub. Health Nutr..

[31] Lusk J.L., Ellison B. (2013). Who is to blame for the rise in obesity?. Appetite.

[32] Pearl R.L., Lebowitz M.S. (2014). Beyond personal responsibility: Effects of causal attributions for overweight and obesity on weight-related beliefs, stigma, and policy support. Psychol. Health.

[33] Seltman H.J. Experimental Design and Analysis. http://www.stat.cmu.edu/~hseltman/309/Book/Book.pdf.

[34] Baron R.M., Kenny D.A. (1986). The moderator mediator variable distinction in social psychological-research—Conceptual, strategic, and statistical considerations. J. Personal. Soc. Psychol..

[35] Sobel M.E. (1982). Asymptotic confidence intervals for indirect effects in structural equation models. Sociol. Methodol..

[36] Horgen K.B., Brownell K.D. (2002). Comparison of price change and health message interventions in promoting healthy food choices. Health Psychol..

[37] Epstein L.H., Dearing K.K., Roba L.G., Finkelstein E. (2010). The influence of taxes and subsidies on energy purchased in an experimental purchasing study. Psychol. Sci..

[38] Giesen J.C.A.H., Havermans R.C., Nederkoorn C., Jansen A. (2012). Impulsivity in the supermarket. Responses to calorie taxes and subsidies in healthy weight undergraduates. Appetite.

[39] Waterlander W.E., Steenhuis I.H.M., de Vet E., Schuit A.J., Seidell J.C. (2010). Expert views on most suitable monetary incentives on food to stimulate healthy eating. Eur. J. Public Health.

[40] Nestle M., Jacobson M.F. (2000). Halting the obesity epidemic: A public health policy approach. Public Health Rep..

[41] Hilbert A., Rief W., Braehler E. (2007). What determines public support of obesity prevention?. J. Epidemiol. Community Health.

[42] Levitsky D.A., Pacanowski C.R. (2012). Free will and the obesity epidemic. Public Health Nutr..

